# Blastocyst Formation Rate and Transgene Expression are Associated with Gene Insertion into Safe and Non-Safe Harbors in the Cattle Genome

**DOI:** 10.1038/s41598-017-15648-3

**Published:** 2017-11-13

**Authors:** Milad Khorramian Ghahfarokhi, Kianoush Dormiani, Ali Mohammadi, Farnoosh Jafarpour, Mohammad Hossein Nasr-Esfahani

**Affiliations:** 1 Division of Biotechnology, Department of Pathobiology, School of Veterinary Medicine, Shiraz University, Shiraz, Iran; 2 Department of Molecular Biotechnology, Cell Science Research Center, Royan Institute for Biotechnology, ACECR, Isfahan, Iran; 3 Department of Reproduction and Development, Reproductive Biomedicine Center, Royan Institute for Biotechnology, ACECR, Isfahan, Iran

## Abstract

Integration target site is the most important factor in successful production of transgenic animals. However, stable expression of transgene without disturbing the function of the host genome depends on promoter methylation, transgene copy number and transcriptional activity in integration regions. Recently, new genome-editing tools have made much progress, however little attention has been paid to the identification of genomic safe harbors. The aim of the present study was to evaluate the effect of insertion site, promoter and copy number of transgene on the production of embryos from cattle fibroblast cells following somatic cell nuclear transfer (SCNT). So, three donor vectors were constructed with *EGFP* gene under control of different promoters. Each vector was integrated into safe and non-safe harbors in the genome using phiC31 integrase. Transgenic clones with a single copy of each vector were isolated. Each clone was analyzed to find site and frequency of integration, expression level and promoter methylation before SCNT, as well as transgene expression level and blastocyst formation rate after SCNT. The data obtained demonstrated that BF5, as a safe harbor, not only showed a stable expression, but also the rate of *in vitro*-produced embryos from BF5-clones are similar to that of non-transfected cells.

## Introduction

Transgenic farm animals such as goats, sheep, and cows are important biomaterials for biomedical and life science researches, including basic research, protein production, and animal models for human diseases^1,2^. The current approaches to the generation of transgenic animals are often inefficient with a low integration rate and variable expression levels due to random and unstable integration of transgene into chromosomal DNA, position effects and the number of inserted copies^1,3^. In order to overcome these defects, the somatic cell nuclear transfer (SCNT) technology has been developed in combination with the site-specific integration of foreign DNA^4^. SCNT technology can effectively increase the efficiency of the production of transgenic farm animals, but cannot overcome the concern about the integration of foreign DNA, which can be resolved by means of phiC31 integration system^2,3^. PhiC31 is a site-specific integrase derived from actinophage QC31 of *Streptomyces* and employed as a powerful genetic tool for efficient non-viral delivery of transgene to the host chromosomal DNA^5,6^. The integrase is a member of DNA recombinase family that encoded within the genome of Streptomyces bacteriophage. This enzyme is functional in mammalian cells thus it can mediate recombination reaction between the *attP* or pseudo-*attP* site in the host genome and *attB* site in the plasmid. This serine integrase can specifically integrate the DNA containing *attB* site into *attP* and produce DNA element flanked by *attL* and *attR* sequences which are no longer identified by phiC31 integrase^2,7^. This system has several useful properties, including unidirectional site-specific integration, stable gene expression, integration into the safe loci within the transcriptionally active area of the host genome with minimum disturbing effect on the function and structure of the target genome^6–8^. Moreover, phiC31 integrase is strictly specific to *attB* sequence (5′-GTGCCAGGGCGTGCCCTTGGGCTCCCGGGCGCG-3′), whereas it does not require conserved *attP* sequence (5′-CCCCAACTGGGGTAACCTTTGAGTTCTCTCAGTTGGGGG-3′) for recombination^5^. On the other hand, this enzyme can mediate the recombination between the *attB* site and the sequences similar to the native *attP* named pseudo- *attP* sites in the genome. These pseudo-*attP* sites are distributed throughout the mammalian genome to such an extent that more than 1000 pseudo-*attPs* have been identified in human, rabbit, rat, mice, cattle, Drosophila, and frog^1,2,5^. Some investigators have reported that pseudo-*attP* sites in the mammalian genome have different rates of integration by phiC31, indicating that the enzyme has different affinities with different pseudo-*attP* sites^2,5,7,9^. Although phiC31integrase inserts the foreign DNA into the safe loci, the function of the DNA transferred to the target genome may be influenced by the position effects^5,10^. In order to overcome this problem, some investigators recommend that applying this system with insulators flanking the transgene cassette maintains a high-level expression^10^. The reduction of transgene expression in the next generations by epigenetic modifications is another important difficulty in transgenic animal production^7,10^. Although the transferred genes are expressed in the transfected cells, they are silenced in the transgenic embryos in preimplantation steps due to the epigenetic changes during reprogramming phenomena, as explained by methylation in the promoter of transgenes^4^. Other factors playing an important role in the fluctuation in transgene expression are CpG motifs and bacterial backbone. Therefore, the depletion of CpG sequences in DNA plasmid can reduce the transgene silencing and improve its long-term expression^11,12^. Genomic safe harbors (GSHs) are genomic locations where integrated transgene have not only the capacity to maintain a stable transgene expression, but no dysregulation in the structure or function of endogenous genes^13^. Adeno-associated virus site 1 (AAVS1) in chromosome 19 and chemokine (C-C motif) receptor 5 (CCR5) gene in human genome, along with Rosa 26 locus in mouse chromosome 6 are the most common genomic sites utilized as the safe harbors^13^. Furthermore, it is reported that the majority of pseudo-*attPs* are located in GSH sites^14–18^. These sites are defined according to the following 5 criteria: (i) far from 5′ end of any gene, (ii) far from the known cancer genes, (iii) far from non-coding RNAs (microRNAs) sequences (iv) outside the transcription units and (v) outside the ultra-conserved regions^13^. According to the above-mentioned criteria, multiple *attP* sites were identified in the cattle genome. BF1, BFF2, BF5, BF12 and BF15 are GSH, while BF4, BF10, BF19 and BF21 are not GSH, since they do not meet 1 or 2 of the above mentioned criteria^3^. Although much progress has been made in genome editing tools, an important obstacle in the generation of transgenic animals is the gradual decline in transgene expression due to epigenetic modifications^10,15^. Based on these complications and the lack of knowledge about GSHs in cattle genome, this study aimed to evaluate the previously described pseudo-*attP* sites in cattle genome for expression level, promoter resistance against de novo methylation, and the effect of integrated region on the reprogramming following SCNT. By employing such sites as robust GSH, it is possible to utilize specific genome-engineering tools such as Crispr/cas9 or TALENs for targeting these regions and the efficient generation of transgenic cattle.

## Results

### Donor vectors and analysis of integration sites in transfected fibroblast cells

**Figure 1:**
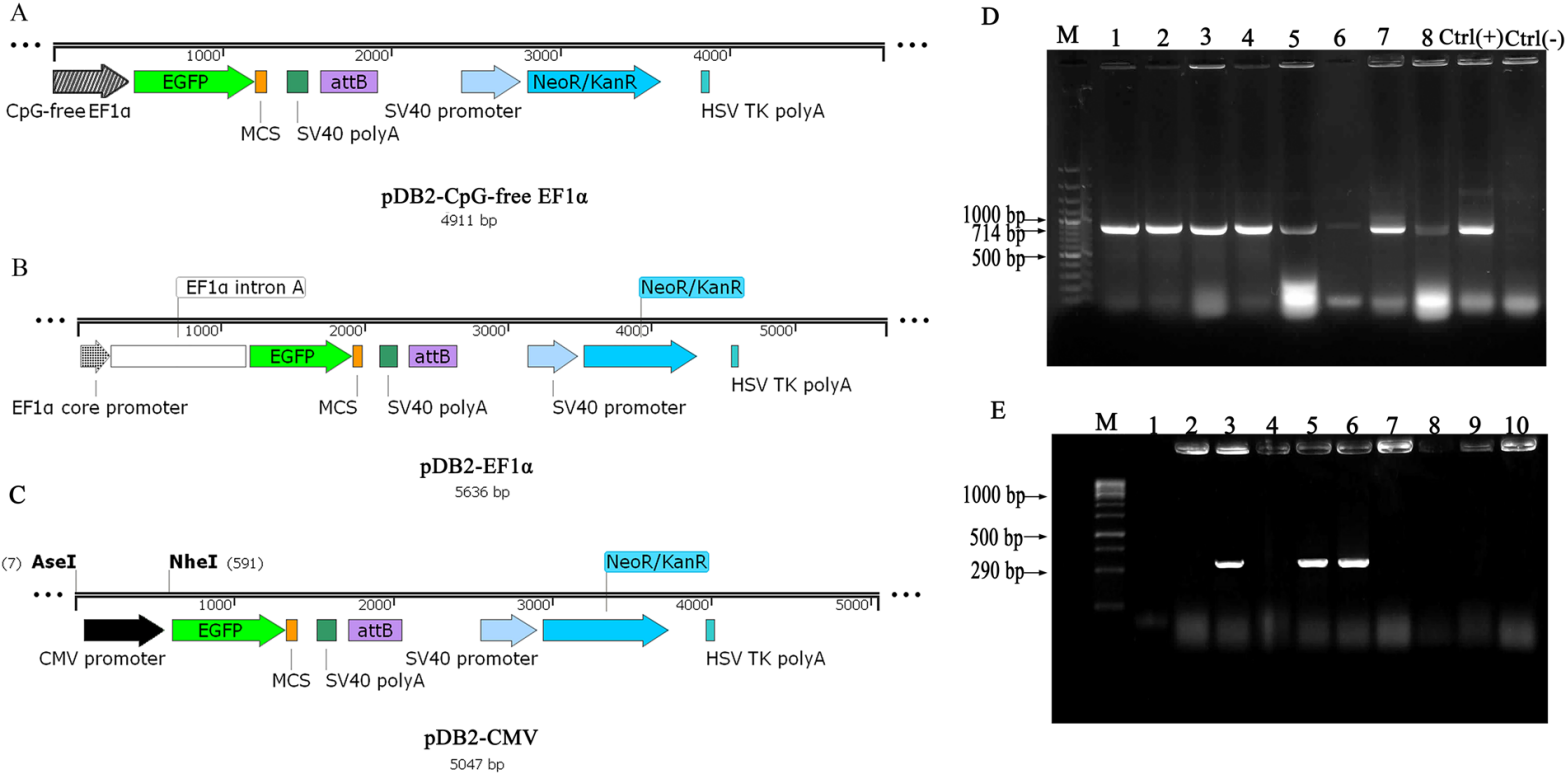
Maps of donor vectors and analysis of specific integration in transfected clones. (**A**) pDB2 vector with CpG-free EF1α promoter. (**B**) pDB2 vector with EF1α promoter. (**C**) pDB2 vector with CMV promoter. The maps were designed by SnapGene 1.1.3 software. (**D**) EGFP coding sequence was amplified in different isolated clones by genomic PCR using EGFPF and EGFPR primers. In lanes 1–8, 714-bp bands confirmed the general integration of the donor vector in recombinant clones. (**E**) The second genomic PCR was designed to confirm the specificity of genomic integration using attBF and attBR primers. The lack of 290-bp band in recombinant clones (lanes 1, 2, 4, 7 and 8) established that phiC31 catalyzed the targeted integration between *attB* and pseudo-*attP* sites. M is DNA ladder 100 bp. Ctrl(+) is positive control in which PCR reaction was performed on pDB2 plasmid and Ctrl(−) is negative control in which PCR reaction was carried out on the genome of non-transfected cells. Electrophoretic gel images presented in this figure were full length.

In addition to pDB2, two donor plasmids with EF1α and CpG-free EF1α promoters were constructed successfully (Fig. 1A–C). The accuracy and correct orientation of cloned fragments was ascertained by sequencing analysis. To test the functionality, endotoxin-free vectors were transfected into primary fibroblasts, which were isolated from cattle skin punch biopsies. The cell imaging of the transfected cells showed the successful expression of fluorescent EGFP in fibroblast cells after 36 h (Supplementary Fig. S1). Two weeks post-transfection of donor vectors along with pCMV-Int into separate groups of fibroblasts, the resistant colonies were emerged using G418 screening. These recombinant cell populations stably expressed EGFP at different levels (Supplementary Fig. S2). These colonies were expanded and genomic DNA samples were harvested for PCR analysis of targeted integration of transgene. In the first genomic PCR, amplification of the expected EGFP band (714 bp) confirmed the integration of donor vector in each examined clone (Fig. 1D). The second PCR was designed to confirm the site-specific integration of the vectors. In this step, PCR bands indicated that the vectors randomly integrated in the genome of some cells. Of the total 62 clones obtained, 12 clones were put aside because the presence of intact 290-bp *attB* band showed that *attB* sequence did not participate in specific integration into the target genome (Fig. 1E).

### Copy number analysis of *EGFP* gene in transfected fibroblast cells

**Figure 2:**
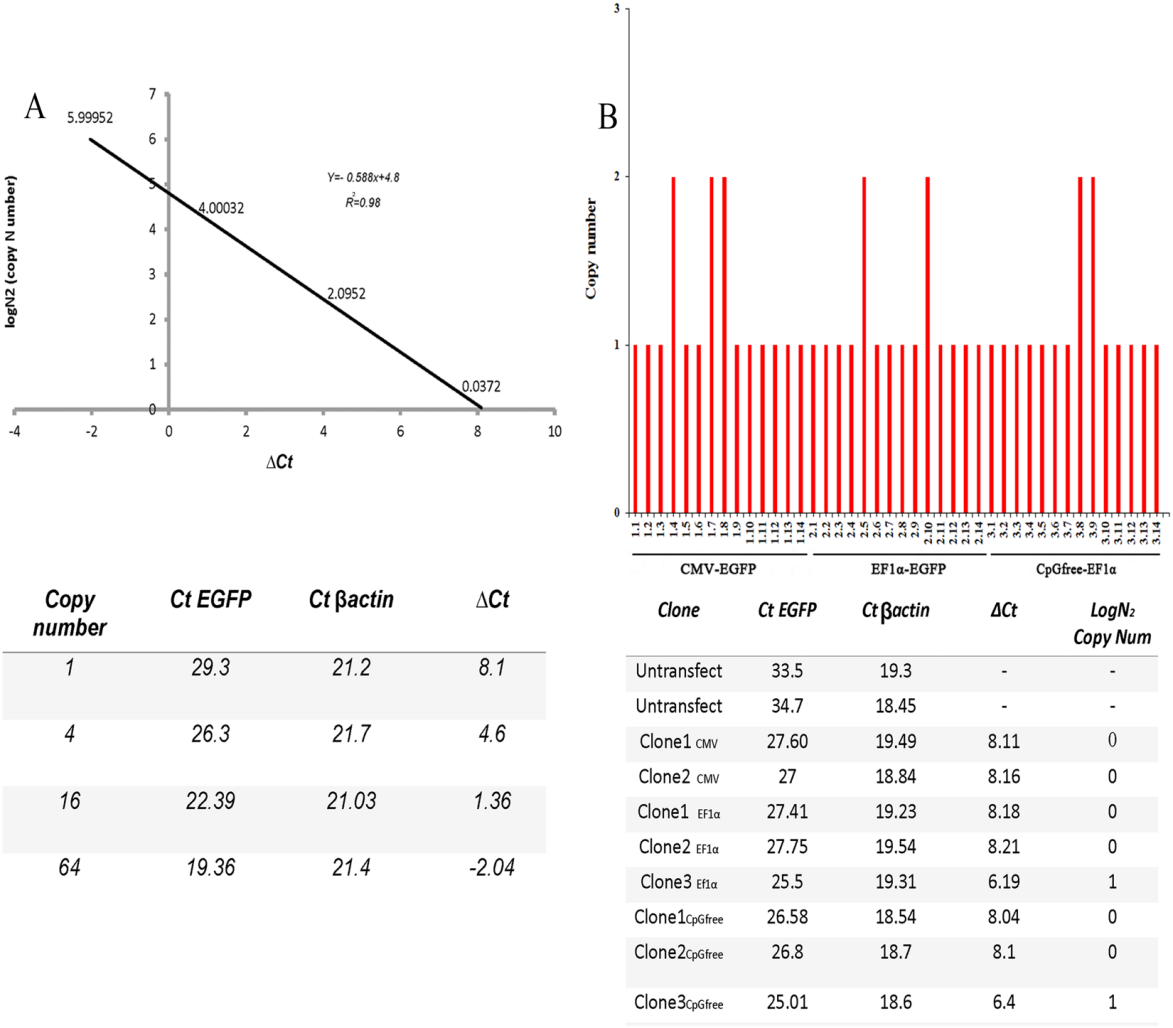
Determination of transgene copies in tested clones. (**A**) The absolute quantitative standard curve based on 1, 4, 16 and 64 copies of donor vector by plotting ΔC_t_ (=C_t EGFP−_  - C_t β-actin_) against log of donor vector copies. R^2^ = 0.98. (**B**) Copy number of donor vector in each transfected clone.

43 out of 50 colonies exhibited single-copy integration, while the remaining colonies contained double-copy integration. The copy numbers were determined by real-time PCR. The absolute quantitative standard curve was drawn by plotting ΔC_t_ against the log of *EGFP* gene copies of the corresponding standard samples (Fig. 2A). The standard curve was determined by linear equation of log_2_^N^ (copy number) = −0.588x + 4.8 (R^2^ = 0.98). We determined the number of EGFP transgene copies in the cells of each transgenic clone by the standard curve and the linear equation (Fig. 2B). Finally, 43 single-copy colonies, including 16 colonies for CMV promoter, 14 colonies for EF1α promoter and 13 colonies for CpG-free EF1α promoter were selected for further analysis (Table 1). The copy number of transgene would drastically affects the expression of the transgene; accordingly we evaluated the transgene expression in the clones with the same copy number of EGFP in order to avoid false results in the transgene expression.

**Table 1:** Integration frequency at pseudo-*attP* sites for different donor vectors.

Vector	Total clones	BF4 site	BF10 site	BF5 site	Others
pDB2-CMV	16	8 (50.00%)	3 (18.75%)	3 (18.75%)	2 (15.50%)
pDB2-EF1α	14	7 (50.00%)	3 (21.42%)	3 (21.42%)	1 (7.14%)
pDB2-CpG-free EF1α	13	5 (38.46%)	4 (30.76%)	3 (23.07%)	1 (7.69%)

### PCR analysis of site-specific integration

**Figure 3:**
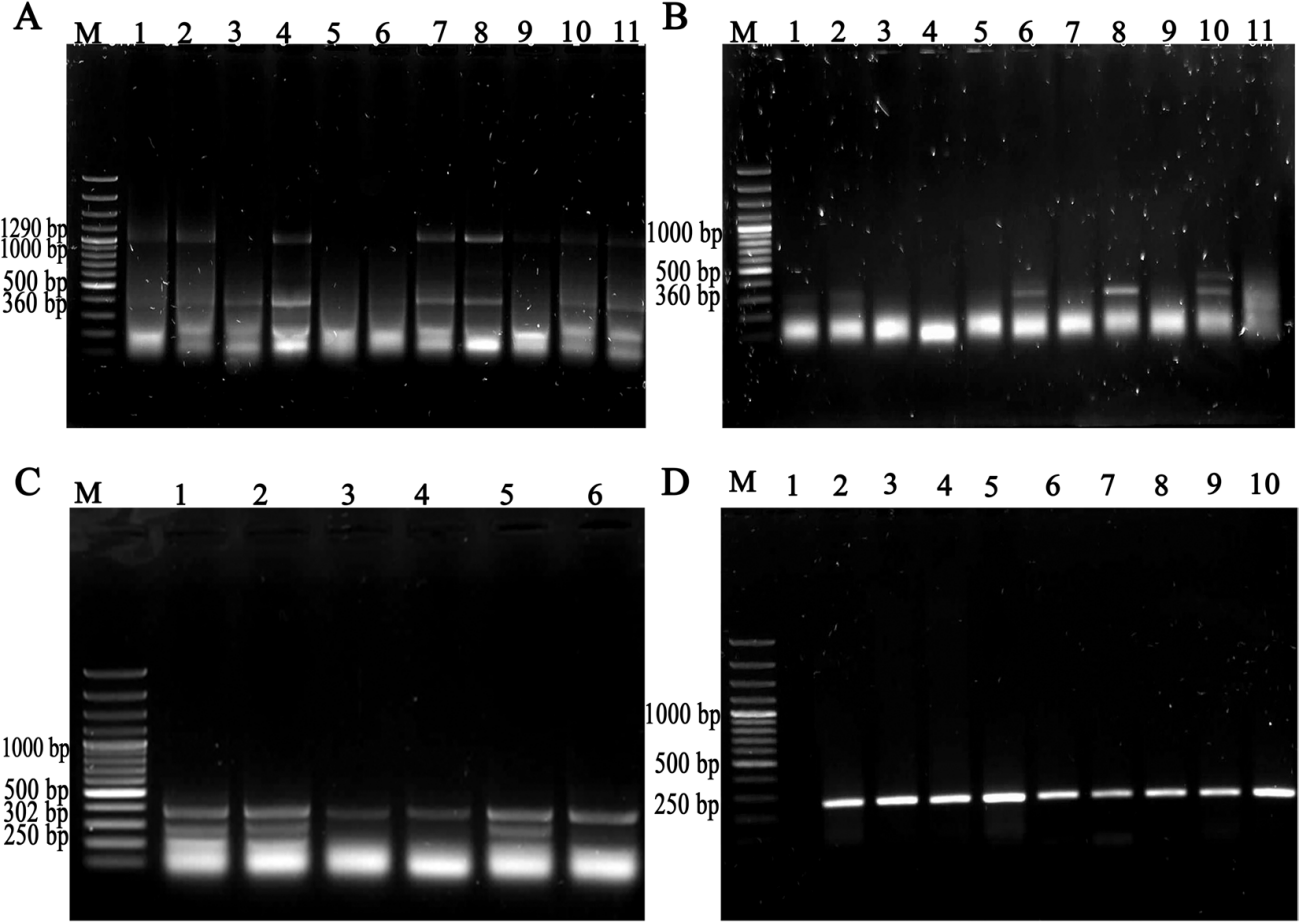
Rescue and analysis of integration site for donor vectors. (**A**) Recombination between *attB* and BF10 pseudo-*attP* sites in the cattle genome was verified using semi-nested PCR. The products were amplified using primers attBF3, 885R and BF10nested. Lanes 1–4, 7, 8, 10 and 11 show the integration of donor vector into BF10 site. (**B**) Recombination between *attB* and BF10 sites was also verified by nested PCR using attBF3 and BF10 nested primers. Lanes 2, 6, 8 and 10 show the integration of donor vector into BF10 site. (**C**) Recombination between *attB* and BF4 pseudo-*attP* sites was verified by semi-nested PCR using attR, attR928L and BF4nested primers. Lanes 1–6 show the vector integration into BF4 site. (**D**) Recombination between *attB* and BF4 sites was also verified by nested PCR using attR and BF4 primers. Lanes 2–10 show the successful integration into BF4 site. M is DNA ladder 100 bp. Electrophoretic gel images presented in this figure were full length.

Identification of vector integration sites in different chromosomes was performed by inverse PCR for BF5 and semi-nested PCR for BF4 and BF10 in 43 clones, which showed single-copy integration of EGFP in the target genome. The 1290 and 360 bp PCR products were obtained for BF10 (Fig. 3A,B) and the 306 and 250 bp bands were achieved for BF4 (Fig. 3C,D)^9^. The location, sequence and integration frequency of each three integration site in the cattle genome were determined and presented in Tables 1,2 and Supplementary Fig. S3. For example, the majority of integration events in 43 single-copy clones were observed at BF4 site located in an intergenic region between the endogenous genes of *GLI3* (upstream of BF4) and *INHBA* (downstream of BF4). Among the clones that were co-transfected with pDB2-CMV and pCMV-Int, the integration rate at BF4 site was higher and encompassed 50% (8 of 16) of the recombinant clones. Furthermore, this vector was integrated into BF10 and BF5 with the same frequency and constituted 18.75% (3 of 16) of the total integration events. The integration frequency at BF4 site with pDB2-EF1α was 50% (7 of 14) of the clones. This vector was integrated into BF10 and BF5 with the integration rate of 21.42% (3 of 14). At last, in the clones which were transfected with pDB2-CpG-free EF1α, the integration frequency at the BF4 site was 38.46% (5 of 13) of the total integration events. The integration rate of this vector at the BF10 was 30.76% (4 of 13) and at BF5 sites was 23.07% (3 of 13) of the total integration events (Table 1). Statistical analysis revealed that the integration frequencies were not significantly different among the utilized constructs.

**Table 2:** Analysis of integration sites as genome safe harbors.

Position and Criteria for GSH	BF5 site	BF4 site	BF10 site
Chromosome	5	4	10
Gene bank accession	NW-001495037	NW-003103903	NW-001492885
Context	intergenic	intergenic	intergenic
Distance to up-stream gene	*RASSF3* 90 kb	*GLI3* 0.2 kb	*FLRT2* 43 kb
Distance to down-stream gene	*TBK1* 100 kb	*INHBA* 186 kb	*LOC1001* 900 kb
At least 50 kb from 5′ end of any gene	✓	—	—
At least 300 kb from cancer related genes	✓	✓	✓
At least 300 kb from any microRNA	✓	✓	✓
Outside of gene transcription unit	✓	✓	✓
Outside of ultra-conserved regions	✓	✓	✓

### Transgene expression from different integration sites of donor cells

**Figure 4:**
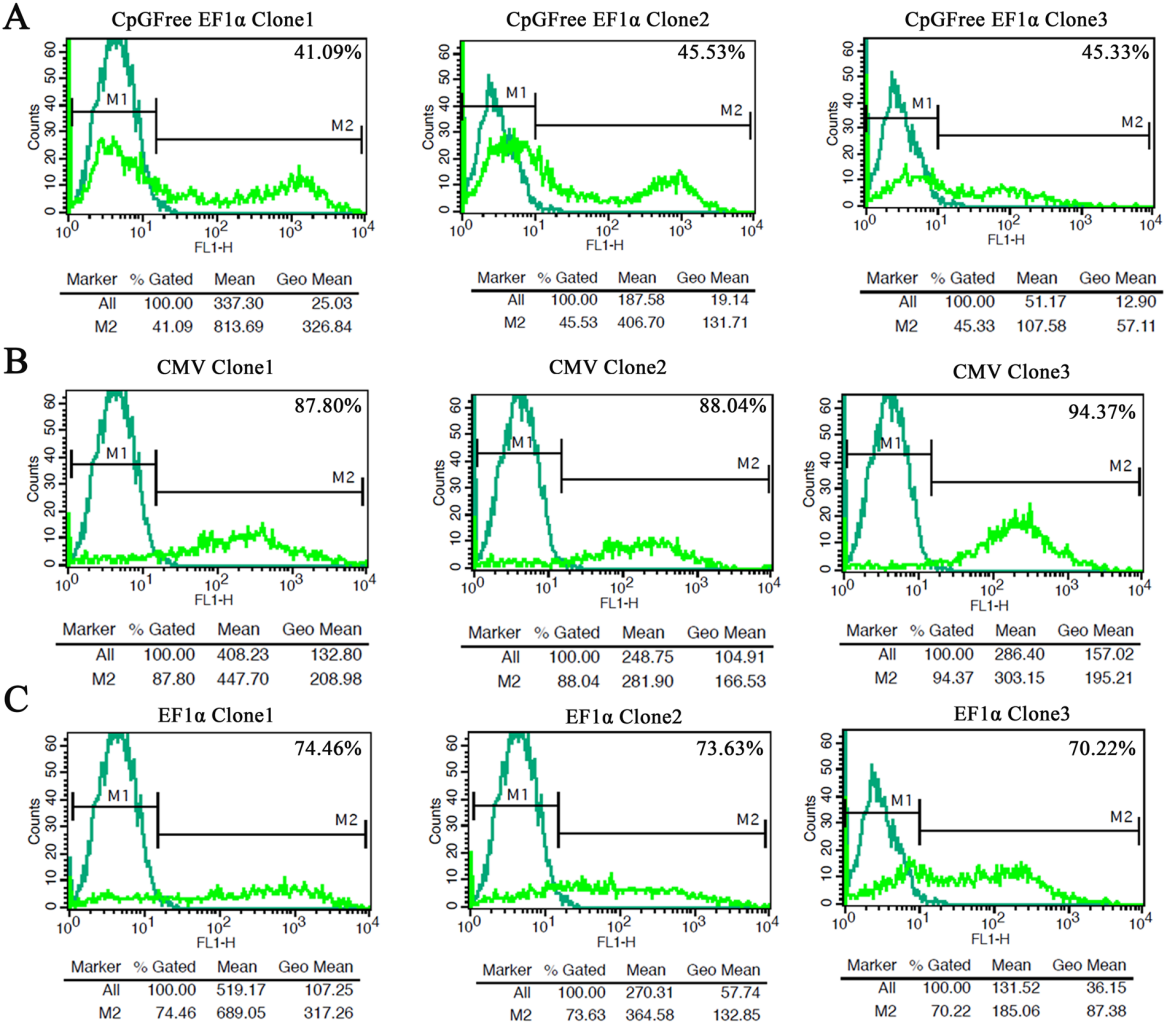
Representative FACS analysis of different transfected clones for expression of EGFP. (**A**) Three transgenic clones contained CpG-free EF1α cassette integrated at BF4, BF10 and BF5 respectively. (**B**) Three transgenic clones contained CMV cassette integrated at BF4, BF10 and BF5 respectively. (**C**) Three transgenic clones contained EF1α cassette integrated at BF4, BF10 and BF5 respectively. Un-transfected fibroblasts were used as a negative control.

**Figure 5:**
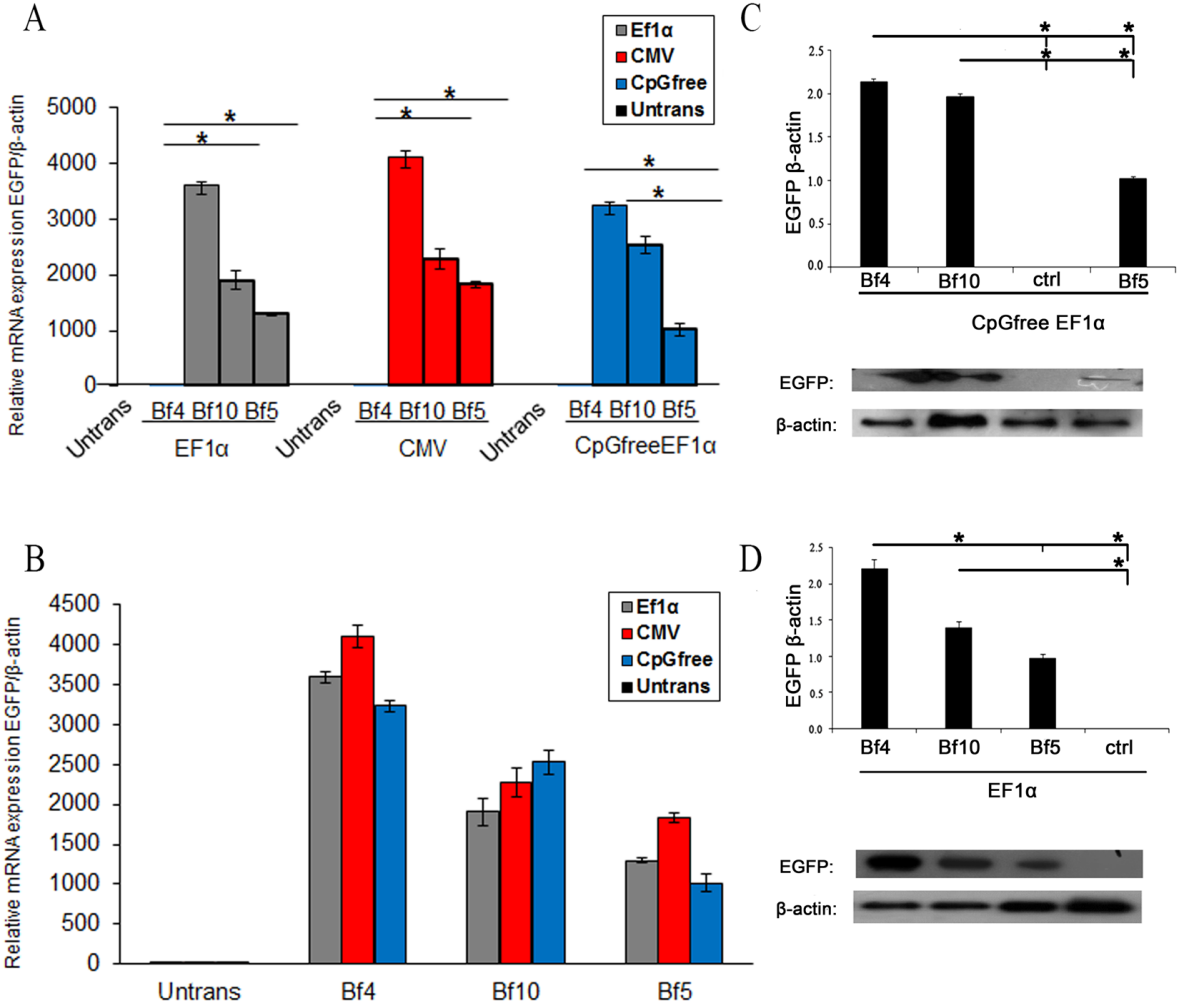
Analysis of EGFP expression in transfected clones contained different integration sites and promoters. (**A**) Relative transgene expression was assessed for all promoters in three different integration sites using RT-qPCR. (**B**) Comparison of relative transgene expression in each integration site by different promoters (*P < 0.05 by ANNOVA and two way ANNOVA). Data presented as mean ± SEM of three separate experiments. (**C**) Western blot analysis for EGFP protein expression under control of CpG-free EF1α promoter in three integration sites. Quantitative analysis of western blot showed significant up regulation in EGFP expression in BF4 and BF10 compare to BF5 site. (**D**) Western blot analysis for EGFP expression under control of EF1α promoter in three integration sites. Quantitative analysis by western blot showed significant up-regulation in EGFP expression in BF4 compare to BF5 and BF10 sites (*P < 0.05 by paired samples t-test). Data presented as mean ± SEM; all reactions were carried out in three separate experiments. Western blot images shown in this figure were cropped; uncropped full length images are shown in the Supplementary Fig. S4.

Fluorescence-activated cell sorting (FACS) was applied to analyze the role of different promoters and integration points on EGFP expression in each cell population used for SCNT (Fig. 4). Flowcytometric analysis of the mean fluorescence intensity (MFI) of the cell population in each transgenic clone indicated a larger increase in fluorescence intensity at the BF4 site compared to the BF10 site (approximately 2-fold) in cattle fibroblasts (Fig. 4A–C). RT-qPCR and western blot were also employed to measure the EGFP expression level by three promoters in different integrated sites of the transfected fibroblasts. The results revealed that all promoters show the highest and the lowest expression levels at the BF4 and BF5 sites respectively (Fig. 5A). A substantial increase in EGFP mRNA level was observed at the BF4 site. This increase was 1.5 to 2-fold more than that of the BF10 and BF5 sites for all promoters (*P* < 0.05), except for the CpG-free EF1α promoter at BF10 site where the transgene expression was more than other promoters (Fig. 5B). These results were further confirmed by western blot analysis for EGFP protein level (Fig. 5C,D).

### Effect of integration site and promoter on EGFP expression in blastocysts

**Figure 6:**
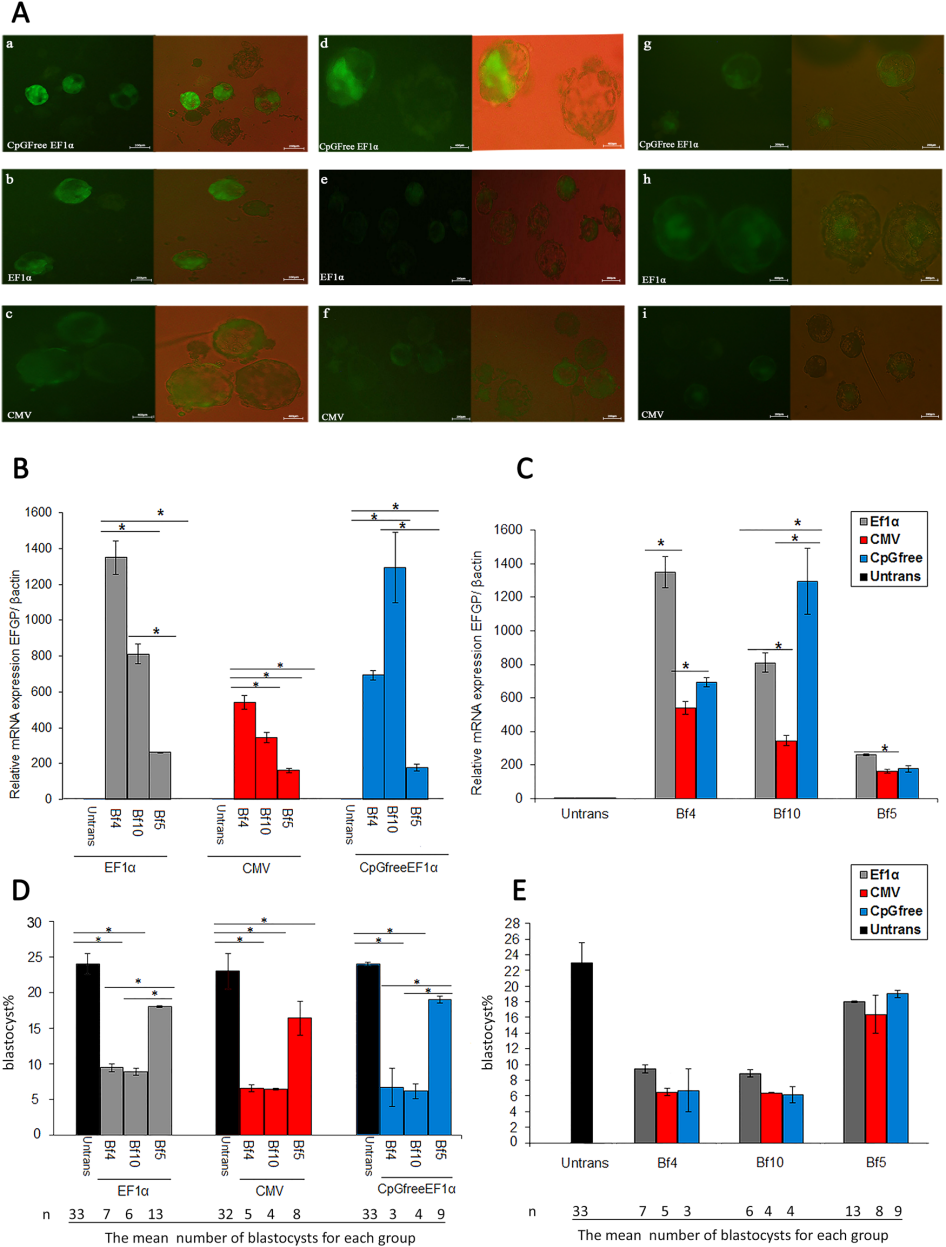
EGFP expression in SCNT-derived blastocysts and blastocyst formation rate from transfected clones contained different integration sites and promoters. (**A**, a–c) Blastocysts obtained by SCNT from BF10-derived clones in which EGFP is under control of CpG-free EF1α, EF1α and CMV promoters. (d–f) Blastocysts obtained by SCNT from BF5-derived clones under control of three different promoters. (g–i) Blastocysts obtained by SCNT from BF4-derived clones under control of three different promoters. Scale bar = 200 and 400 μm. (**B**,**C**) EGFP Expression in three different transgenic clones under control of different promoters. *P < 0.05 means ± SEM of three separate experiments. (**D**) Blastocyst formation rate for any promoter in three different integration sites and the mean number of blastocysts for each group. (**E**) Comparison of blastocyst formation rate in three integration sites by different promoters (*P < 0.05 by ANNOVA and two way ANNOVA). Data are mean ± SEM; all reactions were carried out in triplicate for each clone.

EGFP expression was observed in blastocysts derived from donor cells with different promoters and integration sites using an epifluorescent microscope (Olympus, BX51) (Fig. 6A). EGFP expression was detected by SCNT in blastocysts obtained from the donor cells. For EF1α and CMV promoters, the highest EGFP expression level was observed in BF4-derived blastocysts, so that the transcription level of integrated transgene at the BF4 site was 1.5-fold more than that of BF5 and BF10 sites in blastocysts (*P* < 0.05) (Fig. 6B). Conversely, the CpG-free EF1α promoter showed the highest expression level in BF10-derived blastocysts (Fig. 6C). These results suggest that some CpG motifs in standard EF1α promoter might be methylated. The lowest transcription level was observed in blastocysts derived from transgenic clones containing CMV promoter. Thus, it appears that the EGFP expression was independent of the promoter and only dependents on the integrated site according to above-mentioned results in blastocysts and fibroblast cells, except the BF10 site in blastocysts where the transgene expression was dependent on the type of promoter in addition to the position effect. Overall, the expression of EGFP under the control of three promoters in blastocysts at the BF5 site was moderate unlike the BF4 and BF10 sites (Fig. 6C).

### Effect of integration site and promoter on blastocyst formation

Our results showed that the blastocyst formation rate in clones containing CpG-free EF1α and EF1α promoters at the BF5 site was significantly higher than that of the other two integration sites. However, no significant difference was observed between the BF4 and BF10 sites (Fig. 6D,E). On the other hand, no significant difference was observed between three integration sites in the blastocyst formation from the transgenic clones with CMV promoter (Fig. 6D). The highest rate of blastocyst formation for all promoters was observed in BF5-derived clones (Fig. 6E). Additionally, the blastocyst formation rate in clones containing CpG-free EF1α and EF1α at BF5 site was similar (Fig. 6D,E). The analysis results of *in vitro*-development after SCNT have been demonstrated in Table 3. The blastocyst formation and cleavage rate were not significantly different when BF5 clones were used as nucleic donors compared to untransfected fibroblast cells as negative control. Blastocyst formation rate for EF1α-BF5 clones was 87 ± 1% vs. 90 ± 1.4% and for CpGfree-EF1α-BF5 clones was 91 ± 2% vs. 88 ± 1.78% (*P* > 0.05). Cleavage rate for EF1α-BF5 clones was 18 ± 0.1% vs. 22 ± 2.1% and for CpGfree-EF1α-BF5 clones was 19 ± 0.5% vs. 22 ± 0.4% (*P* > 0.05). Conversely, significant difference was observed between CMV-BF5 clones and untransfected cells. For CMV-BF5 clones, blastocyst formation rate was 15.4 ± 0.65% vs. 21 ± 2.8% (*P* < 0.05) and cleavage rate was 86 ± 1% vs. 91% ± 2.00 (*P* < 0.05) (Table 3).

**Table 3:** Analysis of *in vitro* blastocyst development after SCNT using single-copy clones.

**Donor cell**	**Promoter**	**Insertion site**	**No. culture**	*No cleaved (%)	**No blastocyst (%)
C1.1	CMV	BF4	161	(85% ± 0.10)	(7.0% ± 0.50)
C1.2	CMV	BF10	144	(83% ± 5.50)	(8.8% ± 2.40)
C.1.3	CMV	BF5	146	(86% ± 1.00)	(15.4% ± 0.65)
E1.1	EF1α	BF4	180	(91% ± 4.00)	(8.3% ± 0.55)
E1.2	EF1α	BF5	184	(87% ± 1.00)	(18% ± 0.10)
E1.3	EF1α	BF10	166	(83% ± 4.00)	(8.8% ± 0.45)
CE.1.1	CpG-free EF1α	BF4	118	(95% ± 4.50)	(9% ± 2.70)
CE.1.2	CpG-free EF1α	BF5	125	(91% ± 2.00)	(19% ± 0.50)
CE.1.3	CpG-free EF1α	BF10	158	(93% ± 1.00)	(7.5% ± 1.35)
UC	—	—	164	(90% ± 1.40)	(22% ± 2.10)
UC	—	—	100	(87% ± 1.78)	(22% ± 0.40)
UC		—	86	(91% ± 2.00)	(21% ± 2.80)

UC: Untransfected cells used as a negative control for SCNT experiments.

*$$\mathrm{Cleavage} \% =\frac{\mathrm{Total}-(0\mathrm{PN}+\mathrm{Degenerative})\ast 100}{\mathrm{Total}-\mathrm{Degenarative}}$$

**$$\mathrm{Blastocyst} \% \,=\frac{\mathrm{Blastocyst\; Number}\ast 100}{\mathrm{Total}-(0\mathrm{PN}+\mathrm{Degenerative})}$$.

## Discussion

In this study, we attempted to compare the effect of specific integration sites of phiC31as GSH and non-safe harbor (non-SH) sites on EGFP expression from both donor cells and *in vitro*-derived blastocysts of cattle. This comparison was based on transgene integration rate, transgene expression level and the activity of different promoters in various insertion sites. Moreover, the cleavage rate and the SCNT-derived blastocyst formation rate were evaluated in transgenic clones. Recently, significant progress has been made in specific gene-targeting tools, but it is essential to find and validate predetermined sites as GSHs. Recent studies have indicated that the integration rate of phiC31integrase in non-SHs is more than that in GSHs in human cells^1,3^. According to Yu *et al*., 17 out of 33 phiC31 pseudo-*attP* sites identified by this group in the cattle genome were intergenic and only 5 of them were considered as the GSH site (~15% of the total integration sites)^3^. For example, BFF2 site was introduced as a GSH site in chromosome 2 of the cattle genome^3^. In the present study, we evaluated three integration sites in the cattle genome, including BF4, BF5 and BF10, among which only the BF5 site on the chromosome 5 qualified as a GSH (Table 2 and Supplementary Fig. S3) according to the criteria proposed by Papapetrou *et al*.^3,13^. Also inverse PCR and semi-nested PCR analysis demonstrated that only 9 colonies out of 43 colonies (20.93%) showed integration at the BF5 site located in the intergenic region between *RASSF3* and *TBK1* genes (Tables 1,2). The frequency of site-specific integration in different pseudo-*attPs* are not the same, and is related to the sequence of the site^19,20^. Although the mechanisms underlying this observation are poorly understood, it appears that the selection of pseudo-*attP* sites by integrase depends on the chromosomal context^21^. It is also depends on repetitive sequences near the integration site, which can determine the integration frequency at each hot spot^3^. BF4 and BF10 pseudo-*attP* sites were first reported in 2009 in the cattle genome and it has been shown that the integration of transgene occurred mostly at BF4 site compared to BF10^7,9^. According to our results, BF4 site was preferred to BF5 and BF10 sites for genomic integration; therefore we could not obtain equal number of transgenic clones for each site. Furthermore, our data demonstrated that all constructs were inserted into BF4, BF10 and BF5 with the similar integration rate, but integration frequency was notably different among mentioned pseudo-*attP* sites (Table 1). In a study, Ou *et al*. established that the integration frequency was associated with transgene expression level because some factors are common for both activity such as chromatin accessibility^9^. In the production of transgenic animals, long-term stability of transgene expression is a critical factor. In this regard, loss of foreign gene, position-effect variegation and epigenetic phenomena are among the causes of gradual reduction of transgene expression during SCNT and early embryonic development^4,22^. Besides, it has also been shown that the tandem integration of a transgene can lead to transgene silencing^23,24^. Previous reports have revealed that phiC31integrase inserts one copy of the foreign gene into each pseudo-*attP* site^19,20,25^, although another integration event may take place at a different pseudo-*attP* site^26^. In this study, we obtained 43 colonies with single-copy integration and 7 with double-copy integration following fibroblast transfection. Based on our results, we observed a correlation between the transgene expression level and the copy number in transgenic clones. In the clones contained two copies of transgene, a dramatic increase was observed in EGFP expression level compared to clones containing one copy. So, all of the clones selected for SCNT contained only a single-copy of transgene that facilitated the comparison of expression level independent of the copy number in transgenic blastocysts. PhiC31 integrase can integrate the plasmid containing transgene into specific regions of mammalian chromosomes without epigenetic silencing, resulting in consistent long-term expression^25,26^.

**Figure 7:**
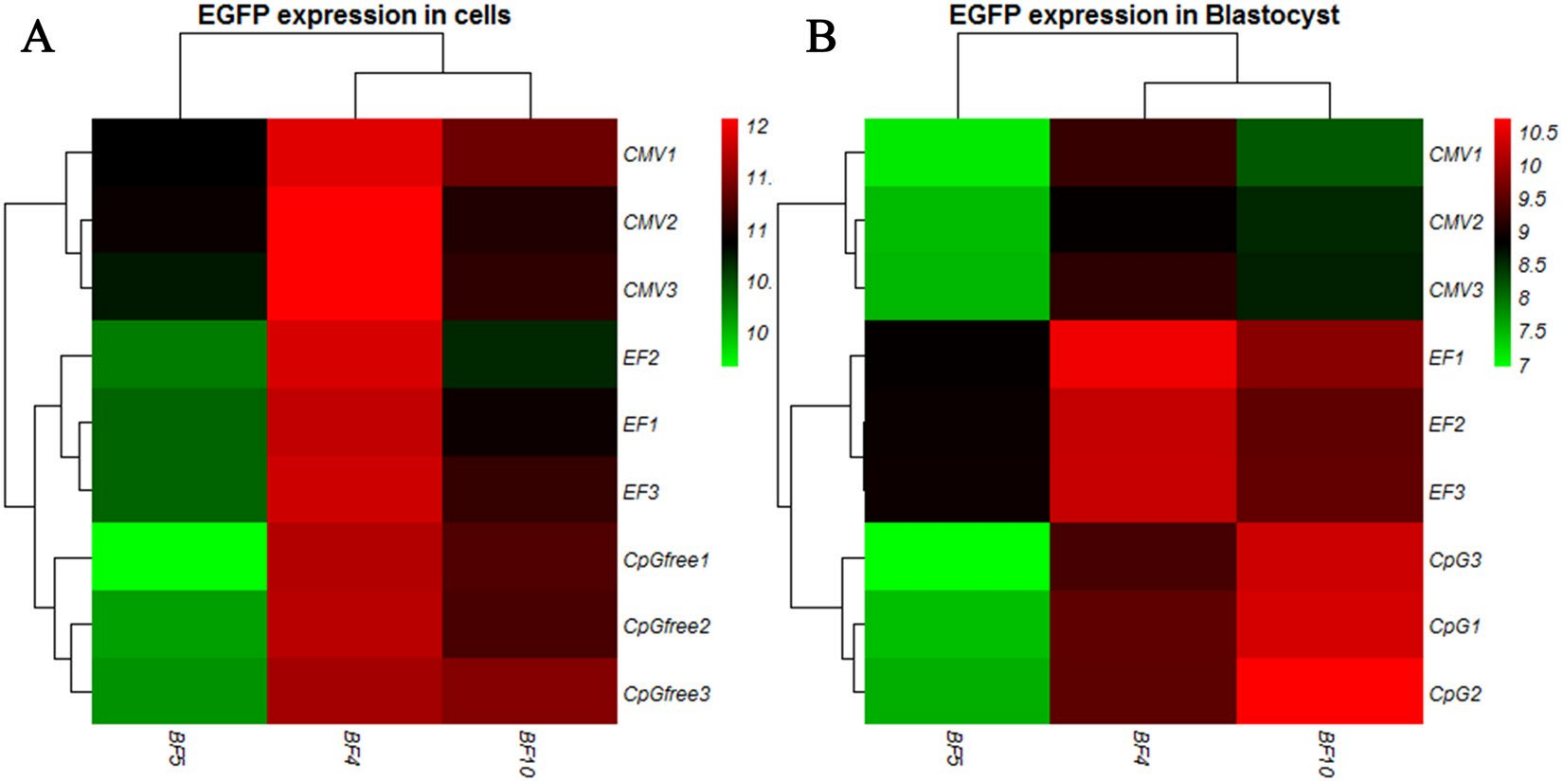
Heat map generated from RT-qPCR data reflecting EGFP expression. EGFP expression was calculated for three promoters from three independent integration sites using custom R script (version 3.4.1). Columns indicate the expression changes at three different integration sites, while rows indicate different promoters for different clones. Color intensity is proportional to relative expressions for each integration site and each promoter, calculated by log_2_^N^ fold change for each clone. Red corresponds to high expression and green corresponds to low expression. EGFP expression data were analyzed by hierarchical clustering for both integration site and promoter. Hierarchical clustering reveals relation EGFP expression across groups. (**A**) Exhibiting different EGFP expression level by three promoters in different genomic sites of donor fibroblasts. (**B**) Exhibiting different EGFP expression level by three promoters in different genomic sites of blastocysts. In each data set, three biological replicates were used for each fibroblast clone and blastocyst group.

Promoter methylation is a powerful cause for transgene silencing *in vitro* and *in vivo* due to methylation of CpG dinucleotide by DNA methyltransferase enzymes. One solution to this problem is the removal of the CpG motifs in the promoter and bacterial backbone, which leads to stable, uniform expression of integrative gene in the genome of target cells^27^. Using CpG-free EF1α promoter, we observed that EGFP expression level was higher than that of wild-type EF1α and CMV promoters from BF10 site in blastocysts (Fig. 6C). However, the expression level was not significantly different among fibroblast donor cells (Fig. 5B). This result proposes that CpG dyads are changed by epigenetic events such as DNA methylation during reprogramming of blastocysts. Our result was in accordance with previous findings, in which a high level of transgene expression was observed when more CGCC sequences were omitted from EF1α promoter^28^. We analyzed and compared the transcriptional activities of three promoters at GSH and non-SH sites as well (Fig. 5A,B). Considering the fact that variation in transgene expression in cattle fibroblasts and blastocysts would generally depends on genomic sites of integration^19,21,29^, we observed that EGFP had a robust expression in BF4-derived clones at both transcription and translation levels (Fig. 5A–D). By comparing different transgene integration sites, our results indicated that expression level at BF4 site was approximately twofold higher than that of BF5 and BF10 sites when the same vector was employed (Fig. 5A). Although the rationale for more expression from BF4 site remained unclear, it appears that the location of this site in 3′UTR of *GLI3* is the main reason for the expression superiority^9^. These observations are consistent with previous studies^7,9^. We also found that transgene expression is independent of the type of promoter in each pseudo-*attP* site of fibroblast cells and it seems that position effect is the main factor affecting the expression level of transgene (Fig. 5A,B). In other words, all promoters showed similar expression efficiency at a distinct site in the fibroblast genome. These findings were confirmed by flowcytometry and western blot analysis (Figs 4 and 5C,D). Our data demonstrated that transgene expression in blastocysts depends not only on the location of genomic integration, but also on the type of promoter employed in BF4 and BF10 sites. But BF5 site showed lower dependency on the type of promoter (Fig. 6C). We also did not observe any significant differences in EGFP expression at the BF5 site in blastocysts with the CpG-free EF1α and CMV promoters (Fig. 6C). However, a slight difference was observed between CpG-free and CpG containing EF1α promoter. In the blastocyst formation, transgene expression dramatically decreased during embryogenesis when CMV compared to other promoters at the BF4 and BF10 sites (Fig. 6B,C). CMV is considered as a powerful promoter and it is broadly used for transgene expression in mammalian cells, however some researchers have proved that this promoter is gradually inactivated after its integration into the host genome, especially *in vivo* ^30^. Likewise, some reports demonstrated that the transcriptional activity is more efficient by EF1α and CBA promoters compared to the CMV in different stages of cell development^31^. According to our results, comparison of EGFP expression profile in various sites with different promoters showed that expression level in non-SH site was higher compared to GSH. In clones that EGFP cassette was integrated in BF5, EGFP expression was much more consistent using different promoters in contrast to non-SH sites in both fibroblasts and blastocysts. Therefore, EGFP expression was higher in SH-sites, but homogeneity of expression was more remarkable in the GSH site. Furthermore, in three recombinant clones for each promoter integrated in a distinct pseudo-*attP* site, we found homogeneity in expression of EGFP (Fig. 7A,B). Transgenic blastocysts derived from the GSH-integrated clones showed a relatively higher developmental rate compared to the non-SH-integrated clones, which might suggest that the integration site of transgene have a critical impact on early embryo development (Fig. 6D,E and Table 3). These results are compatible with the study of Yu *et al*. on BFF2, as a GSH site in cattle genome. They showed that this integration site has a similar cleavage rate, blastocyst formation rate, pregnancy and birth rate to those of the non-transgenic group, whereas the non-SH sites showed a lower developmental rate^3^. However, to our knowledge thus far no study has assessed the transgene expression of different constructs integrated into this site. Our findings demonstrated that blastocyst formation rate, as a critical factor in the generation of transgenic animals, was considerably superior at BF5. Hence, phiC31 integrase is considered as a valuable tool to achieve the stable and efficient expression of a transgene in cells or whole organisms, particularly transgenic animals. Although retroviral vectors have been used to evaluate the expression efficiency of different sites in the mouse genome, but these vectors generally integrated near the transcription start site of the host active genes and CpG island regions with the risk of insertional mutagenesis^32^. In contrast, the pseudo-*attP* sites are generally located in the intergenic and intronic regions of the host genome. The integration of transgene in these sites could prevent disturbances in the function of host genes^33,34^. Another attractive feature of phiC31 enzyme is transgene integration into transcriptionally active, open chromatin domains. This feature leads to a higher integration rate and a more uniform transgene expression^35^. Besides, it is possible to use different promoters in order to regulate transgene expression in different lineages^36^. So, by using phiC31 integrase, it is possible to find and compare multiple transcriptionally active integration sites for finding the new safe harbors in the target genome. When the qualified integration sites are established, they can be employed as the specific targets for genome editing tools such as Crispr/cas9 or TALENs to generate transgenic animals.

In conclusion, we surveyed a number of integration sites of phiC31 enzyme in cattle genome and identified BF5 as a GSH site according to Table 2, which showed higher blastocyst formation rate with moderate transgene expression. The transgenic blastocysts derived from the BF5-integrated clones showed relatively higher developmental competency compared to those obtained from non-SH-integrated clones (BF4 and BF10). This finding suggests that the integration site of transgene has a critical role in the blastocyst formation and consequently production of transgenic animals. Moreover, CpG-free promoters can bypass the epigenetic gene silencing and increase the successful expression of transgene in developing blastocysts. On the other hand, comparison of EGFP expression profile in various sites with different promoters showed a higher expression in non-SH than GSH-integrated clones. We propose that by using of phiC31 enzyme, it is possible to identify new safe harbors among phiC31 integration sites in target genome that can be employed for efficient production of transgenic animals. Also by investigation of the phiC31 integration sites, researcher can determine the possible interference of regulatory elements in neighboring genes on transgenic cassette or potential effects of transgenic cassette on expression of nearby genes.

## Materials and Methods

Unless specified, all chemical and media were obtained from Sigma-Aldrich (St. Louis, MO, USA) and Gibco (Grand Island, NY, USA) respectively.

### Vector construction

The pCMV-Int plasmid containing phiC31 coding sequence and plasmid pDB2 containing *attB* sequence were generously given by Prof. M.P. Calos (Stanford University, USA). For integration of the transgene (EGFP) into the cattle cellular chromosomes, pDB2 plasmid was used. The vector contained an EGFP coding sequence as a reporter gene under the control of the cytomegalovirus (CMV) promoter, a neomycin resistance cassette (*Neo* ^R^) as a selection marker and an *attB* recombination site (Fig. 1C). In addition, two new vectors were constructed that in one of them CMV promoter in pDB2 vector was replaced with an EF1α promoter and in the second it was replaced with a CpG-free EF1α promoter. Details of DNA cloning were presented in SUPPLEMENTARY MATERIALS AND METHODS.

### Primary cell culture

Primary cattle fetal fibroblast (BFF) was isolated and cultured as previously described^37^. Briefly, primary fibroblasts were obtained by trypsinizing a piece of ear skin of fetal cattle. After two times washing of ear skin in PBS containing 50 U/ml of penicillin and 50 mg/ml streptomycin, it was digested with 0.25% trypsin (Sigma-Aldrich, USA) at 37 °C for 1 hour. The skin was minced with scalpel and explanted in 100 mm tissue culture plate containing Dulbecco’s Modified Eagle’s Medium/Ham’s F12 (DMEM/F12) culture media enriched with 10% FBS and then incubated at 37 °C with 5% CO_2_ atmosphere. Twenty-four hours later, 2 ml of media was added to the culture. When the cells reached 70–80% confluency, they were harvested by trypsinization and expanded in T25 flask.

### Transfection

Twenty-four hours before transfection, 2 × 10^5^ BFF cells were added to each well of a 6-well plate (Orange Scientific, Switzerland) contained 2 ml of DMEM/F12 culture medium supplemented with 10% FBS. One well was used as a control group and three remained wells were used for transfection groups. On transfection day, when the cell density of BFF reached 70–80%, the medium was refreshed without antibiotics before cell transfection. Then, 3 µg of pCMV-Int as the integrase-encoding plasmid along with 1 µg of each *attB* donor plasmid (in weight ratio of 3:1) were diluted in Opti-MEM I Reduced Serum Medium. The mixture was employed to stably co-transfect the BFF cells using Lipofectamin LTX (Thermo Scientific, USA) according to the manufacturer’s protocol. At one day post-transfection, 50 U/ml DNase I (Thermo Scientific, USA) was added to the medium in order to remove untransfected vectors. Approximately one-half of the cells in each transfection experiment were seeded into 100-mm culture plate that contained 12 ml of complete cell culture medium and then they were incubated for expansion. The remaining cells were collected and used to measure transfection efficiency by flow cytometry. Details of colony selection were presented in SUPPLEMENTARY MATERIALS AND METHODS.

### DNA extraction and PCR

Total DNA was isolated from transfected calf fibroblast cells at day 35 post-transfection using DNeasy blood and tissue kit (Qiagen, Germany) according to the manufacturer’s protocol. Semi-nested PCR and inverse PCR methods were used to detect three defined integration sites by specific primers for resulted *attL* and *attR* after recombination. The 290-bp band related to *attB* fragment was not amplified due to the recombination between TT core of *attB* sequence in donor vector and pseudo-*attP* site^19^. Semi-nested PCRs were carried out for detection of site-specific integration into BF4 and BF10 sites and inverse PCRs were performed to detect site-specific integration into unknown pseudo-*attP* sites in the genome of transfected fibroblasts before SCNT. BF4 site was detected by semi-nested PCRs using attR928L, attR and BF4nested primers. For detecting the BF10 site, attBF3, 885 R and BF10nested primers were employed (Supplementary Table 1). The details of PCR cycling conditions were presented in SUPPLEMENTARY MATERIALS AND METHODS.

### Gene expression analysis

Total RNA of transfected cell were extracted using Trizol reagent (Sigma, USA) and cDNA synthesis was performed using 1 µg of total RNA in 40 µl volume by random hexamer primers and MMLV reverse transcriptase kit (Takara, Japan). 50 ng of cDNA was employed for RT-qPCR to detect EGFP expression as a reporter and β-actin as a reference gene by CYBR green I (Takara, Japan) using appropriate primers (Supplementary Table 1). All reactions were performed in triplicate in the Thermal Cycler Rotor-Gene 6000 (Corbett, Australia). Vector derived EGFP expression level was measured by the comparative C_t_ method^38^.

### Copy number

Total genomic DNA was isolated from each transfected clone at day 35 post-transfection by using DNeasy blood and tissue kit (Qiagen, Hilden, Germany) according to the manufacturer’s protocol. Standard curve of EGFP target plasmid was generated to measure absolute copy number of *EGFP* gene integrated into the target genome. Series of standard plasmid samples containing 1, 4, 16 and 64 copies of the *EGFP* gene were prepared and mixed with non-transfected of cattle genome according to the following equation; $\frac{{\rm{a}}\times {\rm{b}}\times 0.5}{2.45\times {10}^{9}}$ in which **a** is size of plasmid, 2.45 × 10^9^ is the size of haploid cattle genome^6,39^. The absolute quantitative standard curve was drawn by plotting ∆C_t_ (=C_t EGFP_ − C_t βactin_) against the log of *EGFP* gene copies of corresponding standard samples and it was used to measure the absolute copy number of EGFP target plasmid in each genomic sample.

### Western blot

Protein was extracted simultaneously with RNA using Trizol reagent (Sigma, USA) according to mentioned protocol. 35 µg of each sample was separated by 10% SDS-PAGE and transfer onto polyvinylidene difluoride (PVDF, Bio-Rad, USA) membrane according to standard protocol. Membranes were blocked with 10% w/v skim milk (Merck, Germany) in PBS for 1 hour and then membranes were incubated with primary antibodies, rabbit monoclonal antibody against EGFP (1:100 Abcam) and rabbit monoclonal antibody against β-actin (1:500) in 2% skim milk for 2 hours. Membrane was then incubated with the secondary antibody (HRP-conjugated mouse anti-rabbit IgG (1:16000) and HRP-conjugated goat anti-mouse (1:5000) for 45 min at room temperature. Subsequently membranes were washed in several intervals for 15 min with PBS without Ca^2+^ and Mg^2+^. Finally, HRP signals were detected by Amersham ECL Advance Western Blotting Detection Kit (GE Healthcare, Germany). Chemiluminescence was recorded using UV reader (Uvitec, UK). Densitometric analysis of the bands was performed by imageJ software version 1.4. *β-actin* gene was used as an internal control. The results were subsequently compared by mean relative intensity (mean intensity BF4 band/ mean intensity BF10 or BF5 band). Paired samples t-test was used when two independent groups were compared, the mean difference was significant at the *P* < 0.05 level. Each measurement was performed in replicate.

### Flow cytometric analysis

The mean fluorescence intensity (MFI) of individual transgenic cell populations was estimated using  530/30 nm band pass filter by BD FACSCalibur and data were analyzed with Cell Quest Pro software (Becton Dickinson, USA).

### Screening for transgene expression in recombinant embryos

For RT-qPCR total mRNA were extracted from blastocysts at day 8 of embryo culture using the RNeasy Micro Kit (Qiagen, Hilden, Germany) and subsequently, reverse transcription was carried out to synthesize cDNA using the Prime Script (RT reagent kit, Takara) according to their manufacturer’s recommendation. For reverse transcription, 10 µl of total RNA was added to final volume of 20 µl master mix reaction including 1 µl of hexamer primers, 4 µl of RT buffer (10×), 2 µl of dNTPs, 1 µl of reverse transcriptase and 1 µl of RNase inhibitor (20 IU) (Thermo Scientific, Rochester, NY, USA). Reverse transcription was carried out at 25 °C for 10 min, 42 °C for 1 hour and 10 °C for 10 min. Specific primers (Supplementary Table 1) were used for detection of *EGFP* gene and β-actin as a reference gene quantitative PCR. In this regards, cDNA from SCNT derived embryos were subjected to RT-qPCR with following programs: 94 °C for 5 min as an initial denaturation step, followed by 40 repetitive cycles at 94 °C for 30 s, 60 °C for 30 s, and 72 °C for 20 s. Final extension of 72 °C for 5 min was performed at last stage of PCR. The *β-actin* was used as normalizing gene. Each RT-qPCR was repeated three times. Blastocysts that produced by SCNT and untransfected cattle fibroblast cells were used as negative controls. For each DNA and cDNA sample, one target and reference genes were always amplified independently on the same experimental run in triplicate. The sizes of the amplified products were 140 bp for *EGFP* and 120 bp for *β-actin* (Supplementary Table 1). All reactions were free of primer dimers and non-specific products according to melting curve analysis.

### Oocyte collection, *in vitro* oocyte maturation and production of SCNT embryos

The production of cattle SCNT embryos was carried out as previously described^37,40,41^ by some modifications in enucleation procedure. In brief, cattle ovaries were obtained from a slaughterhouse and transported to the laboratory at 33 °C in saline. Upon receiving the ovaries, they were washed with warmed (37 °C) saline and trimmed. The cumulus oocyte complexes (COCs) were recovered from 2–8 mm follicles using a vacuum pump. Only good quality oocytes were selected and cultured in maturation medium (TCM 199 + 10% FBS (Fetal bovine serum) with 10 µg/ml FSH (follicle stimulating hormone) 10 µg/ml LH (Luteinizing hormone) 100 µg/ml 17β-estradiol, 0.1 nM cysteamine 10 ng/ml EGF (epidermal growth factor) and 100 ng/µl IGF1 (insulin like growth factor 1), and subsequently incubated for 22 hours at 38.5 °C in a humidified 5% CO_2_ atmosphere under mineral oil. Matured oocytes were denuded and zona pellucida was removed by brief incubation in 5 mg/ml pronase. The SCNT procedure was described in details in the supplementary materials and methods.

### Statistical Data Analysis

All data analysis was performed in MS Excel 2007, R packaging version 3.3.3 and SPSS software version 17.0 (SPSS Inc., USA). Experimental data are presented as the mean ± SEM. Statistical analysis of RT-qPCR, western blotting and percentage of blastocyst formation with three independent culture and group. One-way analysis of variance ANNOVA and two-way ANNOVA analysis of variance tests followed by Tukey’s post-hoc test or the paired sample t-test when two independent group adopted for determination of the statistical significance of differences between the proportions. *P* value less than 0.05 was considered statistically significant.

## Electronic supplementary material

Supplementary Information
