# Supplementary material for: Blastocyst Formation Rate and Transgene Expression are Associated with Gene Insertion into Safe and Non-Safe Harbors in the Cattle Genome
Source: Sci Rep. 2017 Nov 13;7:15432. doi: 10.1038/s41598-017-15648-3 (PMC5684190; doi:10.1038/s41598-017-15648-3)
Supplement: Supplementary file 1 — Supplementary Information [file 41598_2017_15648_MOESM1_ESM.pdf]

# Supplementary Information

## Blastocyst Formation Rate and Transgene Expression are Associated with Gene Insertion into Safe and Non-Safe Harbors in the Cattle Genome

Milad Khorramian Ghahfarokhi[1], Kianoush Dormiani[2], Ali Mohammadi[1], Farnoosh Jafarpour[3], Mohammad Hossein Nasr-Esfahani[2,3*]

[1]Division of Biotechnology, Department of Pathobiology, School of Veterinary Medicine, Shiraz University, Shiraz, Iran.

[2]Department of Molecular Biotechnology, Cell Science Research Center, Royan Institute for Biotechnology, ACECR, Isfahan, Iran.

[3]Department of Reproduction and Development, Reproductive Biomedicine Center, Royan Institute for Biotechnology, ACECR, Isfahan, Iran.

[*]Correspondence and requests for materials should be addressed to MH.N.E (email: mh.nasr-esfahani@royaninstitute.org).

## SUPPLEMENTARY MATERIALS AND METHODS

### DAN cloning

Briefly, for EF1α promoter, pBudCE4.1 plasmid (Invitrogen, USA) was digested with *Ase*I and *Nhe*I restriction enzymes and EF1α promoter was isolated and purified by a QIAquick Gel Extraction Kit (Qiagen). CpG-free EF1α sequence was synthesized according to the sequence of CpG-free EF1α promoter in CpG-free vector (Invivogen, USA). Subsequently, the full length of EF1α and CpG-free EF1α promoters were separately subcloned into pDB2 plasmid, which was digested with *Ase*I and *Nhe*I.

### Colony selection

24 hours after transfection, colony selection was started in DMEM/F-12 containing 10% FBS and 500 ng/μl of G418. Selection was continued for two weeks, the resistant clones were picked and each individual clone was expanded for further analysis. The integration rate was calculated according to Thyagarajan *et al*. [20]. The same experiments were performed for all donor vectors containing various promoters (1 μg of each pDB2-CMV, pDB2-EF1α and pDB2-CpG-free EF1α). Two weeks after selection by G418, the cells of individual clones were harvested and divided into three parts, one part was used for genomic DNA extraction, the second part was subjected for EGFP expression analysis by qPCR and the cells in final part were frozen for future SCNT experiments and flowcytometry.

### Semi-nested PCR

The semi-nested PCR round I (PCRI) conditions were as follows: 94°C 5 min as initial step; followed by 32 cycles of 94°C 30 s; 55°C 30 s; 72°C 20 s and a final extension period of 72°C for 5 min. PCR I products were used as template for the second of semi-nested PCR with program as followed: 94°C 5 min as initial step; followed by 35 cycles of 94°C 30 s; 62°C 30 s; 72°C 20 s and a final extension period of 72°C for 5 min. Each amplified fragment was verified by direct sequencing utilizing the appropriate primer. Primers were designed based on available sequences at NCBI (https://www.ncbi.nlm.nih.gov/BLAST/).

### Inverse PCR

Genomic DNA was extracted from cell using DNeasy blood and tissue kit (Qiagen, Germany). 2 μg of each genomic DNA was digested with *Bam*HI (Thermo Scientific, USA) at 37°C followed

by heat inactivation of enzyme at 80°C for 20 min. Digested product was ligated using DNA ligation kit (Takara, Japan) according to manufacturer's protocol. Then, the PCR was carried out using EGFP1 and attBF3 primers as follow: 95°C 3 min; 32 cycles of 95°C for 30 s; 60°C for 30 s; 72°C for 8 min and one step of 72°C for 10 min. Finally, PCR product was cloned into pTZ57RT cloning vector (Thermo Scientific, USA) and sequenced with forward and reverse M13 primers by using automatic DNA sequencing method. All obtained DNA sequencing data were aligned with the *Bous Turus* genome in Genome Browser, University of California Santa Cruz using BLAT tool (https://genome.ucsc.edu).

**SCNT procedure**

Procedure of SCNT was carried out using manual enucleation using a fine pulled Pasteur pipette [41]. In brief, MII oocytes were vortexed with 300 IU/ml hyaluronidase for 3 min for removal of cumulus cells. For removing zona pellucida, denuded oocytes were treated with 5 mg/ml pronase. The method of manual enucleation was used as described previously [41]. Briefly, zona free oocytes were incubated in TCM199 supplemented with 4 µg/ml demecolcine for 1 h in 38.5°C. After that, cytoplasmic protrusion containing MII spindle, was removed by hand-held manual oocyte enucleation pipette. For nuclear transfer, nucleus-free bovine oocytes that have been successfully enucleated were transferred to dishes containing a droplets of H-TCM199 supplemented with 10 mg/ml phytohemagglutinin, and a well-rounded bovine fibroblast (un-transfected or transfected) cells were attached to membrane of enucleated oocytes. Subsequently couplets were electrofused in fusion buffer without $Ca2^{+}$ and $Mg2^{+}$. After 30 min, reconstructed oocytes were activated with 5µM Ca-ionophore for 5 min followed by incubation with 2 mM 6-dimethylaminopurine for 4 h. Then, activated reconstructed oocytes were cultured in modified synthetic oviductal fluid (mSOF). Embryos were cultured in group of six in wells drained with a fine pin in 20 µ1 of mSOF under mineral oil at 38.5°C, 5% $CO_2$, 5% $O_2$ and humidified air for 7 days.

## SUPPLEMENTARY FIGURES LEGENDS

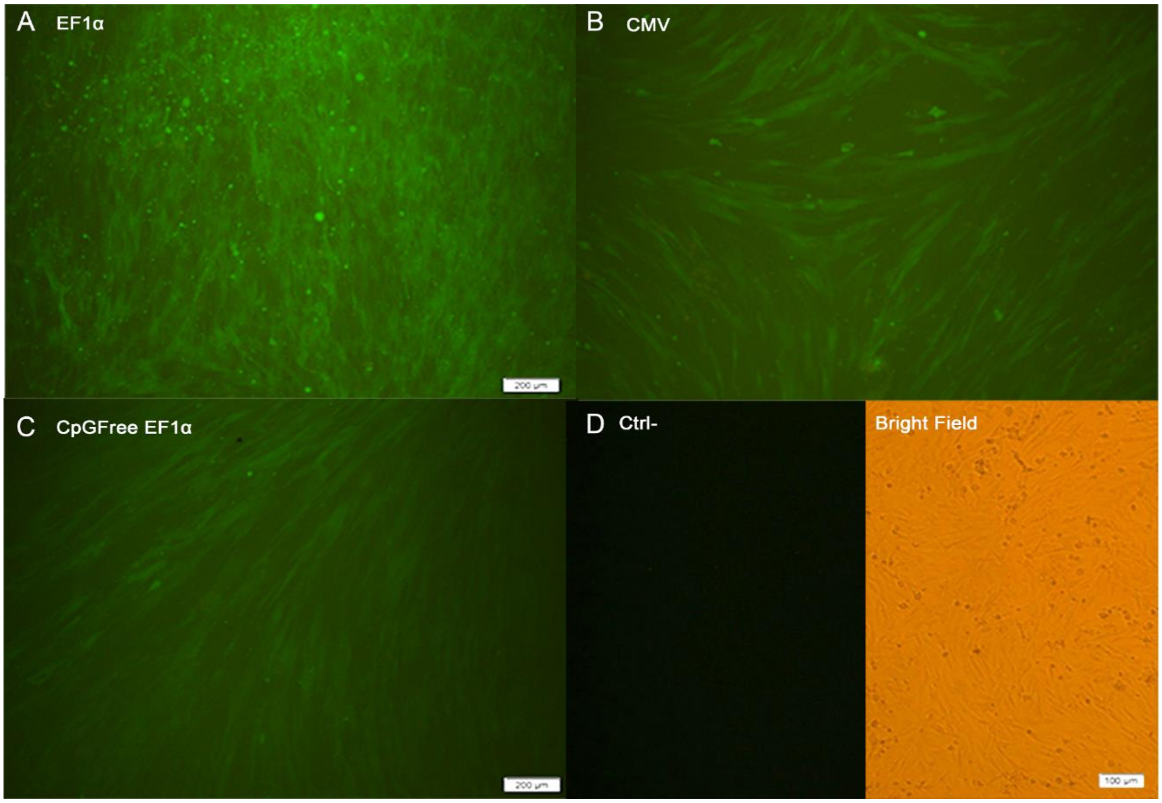


**Supplementary Figure S1. Fluorescent analysis of three separate groups of fibroblast cells 36 hours post-transfection.** (**A**) Transfected cells by pDB2-EF1α. (**B**) Transfected cells by pDB2-CMV. (**C**) Transfected cells by pDB2-CpGfree EF1α. (**D**) Untransfected cells as the negative control. Scale bar = 200 µm.

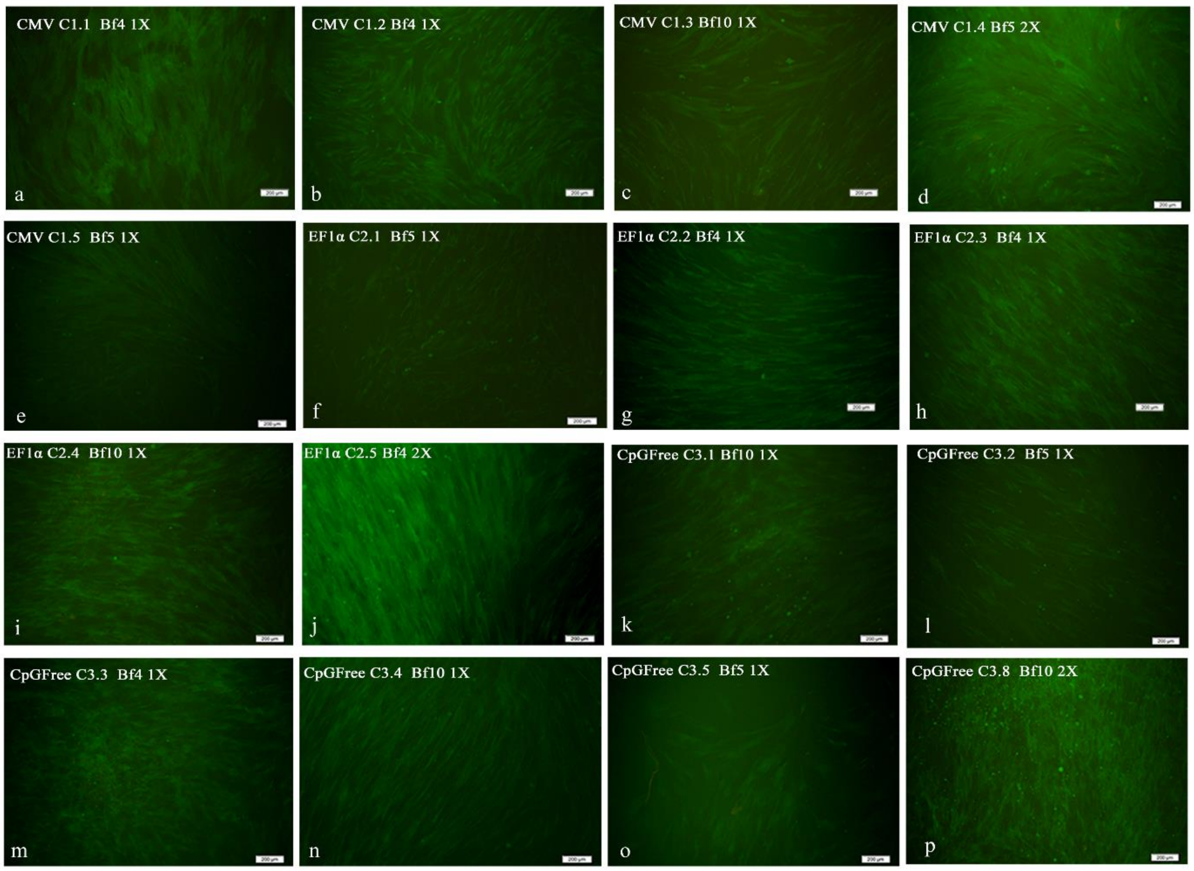


**Supplementary Figure S2. Fluorescent images of fibroblast cells co-transfected with different donor vectors along with pCMV-Int.** Transfected clones contained one or two copies of EGFP gene that were isolated after two weeks using G418. **a, b, c** and **e** are the clones with one and **d** is a clone with two copies of EGFP gene, all under control of the CMV promoter. **f, g, h** and **i** are the clones with one and **j** is a clone with two copies of EGFP gene under control of EF1α promoter. **k, l, m, n** and **o** are the clones with one and **p** is a clone with two copies of EGFP gene under control of CpG-free EF1α promoter. In **a, b, g, j** and **h-m** clones, integration was carried out in BF4 site and in **c, i, k** and **n-p** clones, integration was performed in BF10 site. In **e, f, o** and **d-l** clones, donor vector was integrated into BF5 site. Scale bar = 200 µm.

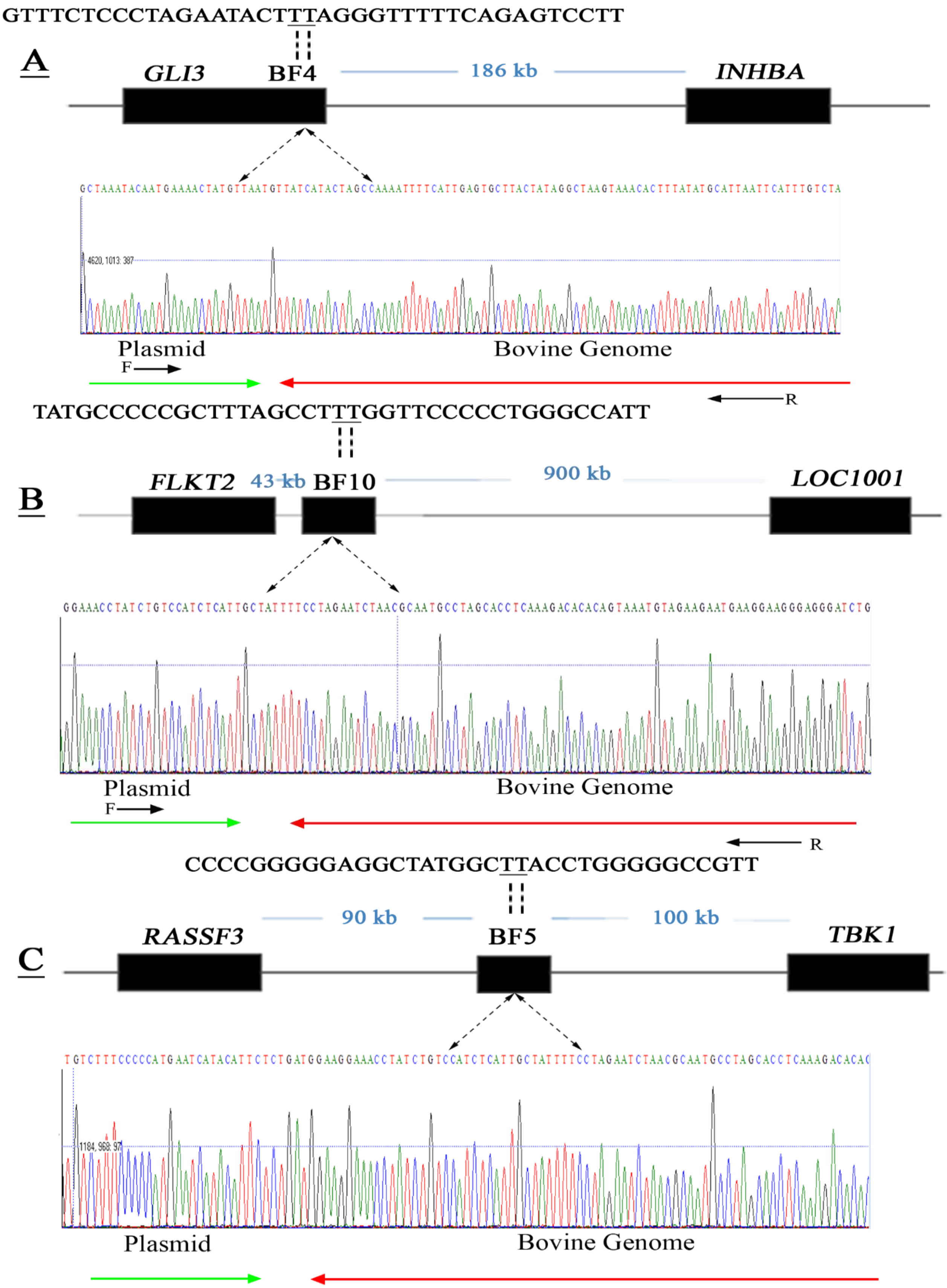
GTTTCTCCCTAGAATACTTTAGGGTTTTTCAGAGTCCTT
A
GLI3
BF4
186 kb
INHBA
Plasmid
Bovine Genome
F
R
TATGCCCCCGCTTTAGCCTTTGGTTCCCCCTGGGCCATT
B
FLKT2
43 kb
BF10
900 kb
LOC1001
Plasmid
Bovine Genome
F
R
CCCCGGGGGAGGCTATGGCTTACCTGGGGGCCGTT
C
RASSF3
90 kb
BF5
100 kb
TBK1
Plasmid
Bovine Genome

**Supplementary Figure S3. The location and sequence of each integration site in the cattle genome.** (**A**) BF4 integration site was detected by PCR using F and R primers. It is located 186 kb upstream of *INHBA* gene and downstream of *GLI3* in 3´UTR. (**B**) BF10 integration site was detected by PCR using F and R primers. It is located 900 kb upstream of *LOC1001* gene and 43 kb downstream of *FLKT2* gene. (**C**) BF5 site is located 100 kb upstream of *TBK1* gene and 90 kb downstream *RASSF3* gene.

**Supplementary Table 1: List of primers used for amplification, nested PCR, inverse PCR and RT-qPCR.**

| Primer | Sequence | Annealing (°C) | PCR product |
|---|---|---|---|
| **†attR** | 5'- GGATCAACTACCGCCACCT- 3' | 60 | 302 bp |
| **†attR928L** | 5'-GCTGGACGTGTAACCCCTTA-3' | | |
| **†attR** | 5'- GGATCAACTACCGCCACCT -3' | 58 | 250 bp |
| **BF4 nested** | 5'-TGGAATAACGGAGAGACACG-3' | | |
| **† attBF3** | 5'- GTAGGTCACGGTCTCGAAGC -3' | 58 | 1290 bp |
| **†885R** | 5' TTG ATA CAC AGC CTC GCT TG 3' | | |
| **†attBF3** | 5'- GTAGGTCACGGTCTCGAAGC -3' | 62 | 360 bp |
| **BF10nested** | 5'-TCCTCACGATTTGCACACTG-3' | | |
| **attBF** | 5'-GTCGACGATGTAGGTCACG-3' | 62 | 290 bp |
| **attBR** | 5'-ATGCCCGCCGTGACCGTCGAGA-3' | | |
| **rGFPF** | 5'-CAAGCAGAAGAACGGCATCAAG-3' | 61 | 145 bp |
| **rGFPR** | 5'-GGTGCTCAGGTAGTGGTTGTC3' | | |
| **βactinF** | 5`-TCGCCCGAGTCCACACAG-3` | 59 | 200 bp |
| **βactinR** | 5`-ACCTCAACCCGCTCCCAAG-3` | | |
| **GFP complete F** | 5`-ATGGTGAGCAAGGGCGAGGAG-3` | 60 | 714 bp |
| **GFP complete R** | 5`-ATTACTTGTACAGCTCGTCCATG-3` | | |

† These primers have been used for detection of integration sites by Ou *et al.* [9].

## UNCROPPED GEL IMAGES

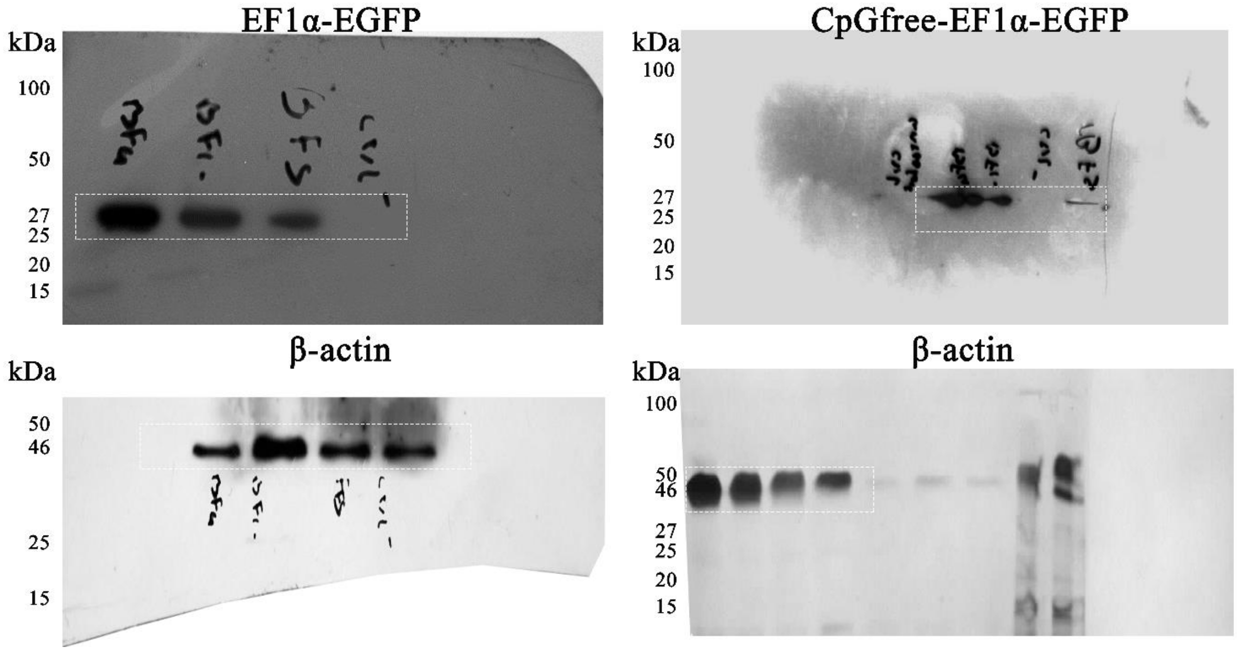


**Supplementary Figure S4. Uncropped full length western blot images of EGFP and β-actin.** The images were cropped in dotted boxed regions and presented in the Fig. 5C,E of the main text.
